# Supplementary material for: NK2R signaling governs intestinal lipid mobilization and mucosal inflammation
Source: eLife. 2026 Oct 5;15:RP109903. doi: 10.7554/eLife.109903 (PMC13638200; doi:10.7554/eLife.109903)
Supplement: Peer review history

# Editor report: eLife Assessment

**DOI:** 10.7554/eLife.109903.2.sa0

**Authors:** Ana Maria Faria

**Affiliations:** Universidade Federal de Minas Gerais Brazil

**Keywords:** Important, Incomplete, Solid

This is an **important** study on the role of the neurokinin-2 receptor (NK2R) as a regulatory node connecting intestinal lipid metabolism, mucosal immunity, and the gut microbiome, bidirectionally regulating enterocyte lipid uptake, lipid droplet storage, chylomicron output, and systemic metabolic parameters in DIO mice. The authors present **solid** evidence linking Tacr2 deletion to reprogrammed epithelial lineage allocation, dampened immune gene expression, and male-biased protection from DSS colitis, despite dysbiotic microbiota. However, the causal evidence for some mechanistic and pro-inflammatory NK2R claims remains **incomplete** and potentially confounding, requiring additional cell-type-specific and functional experiments.

# Referee report: Reviewer #1 (Public review)

**DOI:** 10.7554/eLife.109903.2.sa1

Summary:

This study identifies NK2R as an intestinal GPCR that tunes enterocyte lipid uptake, lipid droplet storage, and chylomicron output, with loss or antagonism enhancing post‑prandial triglyceridemia and epithelial lipid stores, and agonism reducing adiposity and improving glycemia in DIO mice. Through bulk RNA‑seq, deconvolution, DSS colitis, and 16S profiling, the authors link Tacr2 deletion to coordinated induction of epithelial lipid‑metabolic programs, dampened immune gene expression, sex‑specific remodeling of secretory lineages, and male‑biased protection from experimental colitis despite dysbiotic microbiota. This is an overall important and thorough paper on an emerging obesity drug target, but it should temper some interpretations, and the following points would be needed to strengthen the claims in the manuscript.

Strengths:

The study uses an impressive combination of genetic loss‑of‑function, pharmacological agonism/antagonism, transcriptomics, and in vivo physiology to establish NK2R as a bidirectional regulator of epithelial lipid handling. The integration of RNA‑seq, epithelial cell‑type deconvolution, DSS colitis, and microbiome profiling provides a rich, systems‑level view of how Tacr2 deletion reshapes epithelial metabolism, lineage allocation, and inflammatory responsiveness in a sex‑specific manner. The gain- and loss‑of‑function data particularly support a model in which NK2R acts as an epithelial metabolic rheostat that restrains lipid absorption and chylomicron export, with downstream consequences for barrier fitness and immune tone.

Weaknesses:

Major points

While the data convincingly establish NK2R's role in epithelial lipid handling, the manuscript arguably overstates a primary "pro‑inflammatory" function for NK2R, given that Tacr2‑/‑ mice show enhanced enterocyte lipid uptake and storage, higher post‑prandial triglycerides, and a dysbiotic microbiota yet reduced mucosal immune gene expression and, in males, protection from DSS colitis. It remains equally plausible that the apparent "protection" reflects a mucosa that is less reactive to unfavorable microbiota rather than genuinely protected, and that NK2R's main function is metabolic, with immune changes emerging secondarily. Such a model would actually help reconcile the long-standing question as to why NK2R antagonism has not translated into clear benefit in clinical trials for GI inflammation over the past several decades.

Without temporal resolution, it is equally plausible that antagonists primarily perturb epithelial lipid homeostasis rather than directly and beneficially modulating immune tone. To discriminate between these possibilities and strengthen the potential direct inflammatory claims, the authors should:

(1) generate epithelial‑specific, immune‑cell-specific, and nociceptor‑specific Tacr2 deletions in the DSS model

(2) test gut‑restricted NK2R agonism versus antagonism under controlled dietary fat conditions for effects on LD load, barrier integrity, and colitis severity

(3) perform ex vivo tachykinin/NK2R stimulation of isolated epithelial versus immune compartments with functional readouts

(4) assess whether microbiota transfer from Tacr2‑/‑ versus WT donors into germ‑free or antibiotic‑treated recipients can recapitulate protection or susceptibility independently of epithelial NK2R status.

Minor points

Additional clarifications on Tac1 and tachykinin receptor expression in male/female colitis models, and validation of the NK2R antibody in KO tissue (or in situ hybridization), would also be needed to strengthen key mechanistic and localization claims.

# Referee report: Reviewer #2 (Public review)

**DOI:** 10.7554/eLife.109903.2.sa2

Summary:

This manuscript- "NK2R signaling governs intestinal lipid mobilization and mucosal inflammation" by Perez et al investigates the role of the neurokinin-2 receptor (NK2R) as a regulatory node connecting intestinal lipid metabolism, mucosal immunity, and the gut microbiome. The authors utilized a ubiquitous deleted Tacr2 mouse model alongside targeted pharmacological treatments to demonstrate that NK2R limits luminal lipid uptake and chylomicron secretion. Additionally, the study uncovers that Tacr2 deficiency promotes male-biased protection against DSS-induced colitis and drives distinct diet- and genotype-dependent shifts in the fecal microbiota.

Strengths:

(1) The authors successfully utilized both a genetic whole-body knockout model (Tacr2-/-) and targeted pharmacological agents, such as the antagonist GR159897 and the agonist EB1002. This dual approach effectively corroborates the core phenotypic findings.

(2) The study provides a compelling case for targeting the tachykinin-NK2R axis therapeutically. The remarks that NK2R agonists could be leveraged to treat obesity, while antagonists might be used for inflammatory bowel disease, will be an exciting clinical outcome if further validated.

(3) The integration of RNAseq for epithelial lineage analysis, combined with in vivo gut permeability assays, lipid tolerance assays, and 16S microbiome sequencing, provides a robust and highly detailed physiological picture.

Weaknesses:

This manuscript has some notable limitations. While the transcriptomic data show an upregulation of the enterocyte lipid droplet program in Tacr2-/- mice, the manuscript lacks biochemical experiments to conclude the downstream signaling mechanism driving such changes. The reliance on a global whole-body knockout model confounds the ability to definitively conclude that the observed metabolic and inflammatory phenotypes are linked to the intestinal epithelium. The authors discuss a male-biased protection against DSS-induced colitis, but they rely on speculation regarding sex hormones rather than providing experimental data to explain this dimorphism.
